# Agreement Between 2D and 3D Echocardiography in Measuring Dimensions of the Patent Ductus Arteriosus in Infants

**DOI:** 10.1007/s00246-025-03856-y

**Published:** 2025-05-22

**Authors:** Stephan Juergensen, Christine Springston, Michael J. Bunker, Phillip Moore, Shafkat Anwar

**Affiliations:** 1 Division of Cardiology, Department of Pediatrics, Columbia University Vagelos College of Physicians and Surgeons and New York Presbyterian-Morgan Stanley Children’s Hospital, New York, NY USA; 2 Division of Cardiology, Department of Pediatrics, University of California San Francisco, San Francisco, CA USA; 3 Center for Advanced 3D+ Technologies, University of California San Francisco, San Francisco, CA USA

**Keywords:** Pediatric echocardiography, Patent ductus arteriosus, 3D echocardiography, Congenital heart disease, Procedural planning

## Abstract

The patent ductus arteriosus (PDA) is a common cardiac lesion in neonates, which may require intervention for patency, or closure in the neonatal period. Two-dimensional echocardiography (2DE) is standard for PDA imaging. Three-dimensional echocardiography (3DE) is increasingly used for assessing complex anatomy and pre-procedural planning; however, there are limited data on the value and accuracy of 3DE of the PDA in infants. We aimed to determine the degree of agreement between 2 and 3DE in measuring common dimensions of the PDA in infants. Infants < 1-year-old, > 32 weeks corrected gestational age (CGA), and > 1 kg with a known PDA were enrolled prospectively for imaging by 2DE and 3DE. Images were collected at the parasternal short axis (PSAX) and suprasternal notch (SSN). Dimensions were measured at the pulmonic (PA) and aortic (Ao) end. Interclass correlation (ICC), Bland–Altman (BA), and coefficient of variability (COV) assessed interobserver variability. 2DE to 3DE association was assessed by BA. Twenty-nine subjects were enrolled and had complete data sets, median CGA 39.4 weeks (IQR 38.3–41.7), median weight 3.250 kg (IQR 2.870–4.295). ICC for all 2D and 3D images in PSAX and SSN views was strong with narrow limits of agreement (LOA). BA showed low bias and generally narrow LOA. Each observer’s 2DE- 3DE comparison yielded low bias and narrow LOA. There was generally no statistically significant difference in PDA size when 2D and 3D images were compared for each observer. Our data suggest 2DE and 3DE have strong agreement and paired 2DE- 3DE images show low bias and limited variability for common PDA measures. Previous work in this area has focused on larger patients, and this data begins to build a foundation for use of 3DE for anatomic assessment and possible interventional planning in smaller patients with PDAs. Further study should aim to compare 3DE agreement with CT or angiography, and at variable subject size, ductal size, and ductal type.

## Introduction

The ductus arteriosus, a necessary fetal vascular structure connecting the aorta and pulmonary arteries, generally closes shortly after birth as a part of transition of the fetal to postnatal circulations. Persistence of the ductus arteriosus, known as patent ductus arteriosus (PDA), is a common and often clinically important cardiac anomaly in neonates. In a structurally normal heart, a PDA can lead to volume overload of the pulmonary vascular bed, leading to pulmonary overcirculation, left heart dilation, congestive heart failure, and decreased systemic circulation. These issues are particularly amplified in premature infants, who have a 20%–60% incidence of persistent PDA [1]. Well-documented risks associated with persistent hemodynamically significant PDA in premature and young infants include necrotizing enterocolitis, pulmonary hemorrhage, intraventricular hemorrhage, poor growth, and increased overall mortality [1–5]. In infants with congenital heart disease, a PDA may be necessary to maintain systemic or pulmonary blood flow prior to surgical intervention and thus must be maintained. With expansion in device options and interventional techniques, neonates and infants are increasingly having these closed in the catheterization lab as opposed to the operating room, and PDA stenting is increasingly used to maintain an external source of pulmonary blood flow in children with critical pulmonary outflow obstruction. In either instance of closure or maintained patency, a thorough understanding of the anatomy and dimensions of the PDA is needed for interventional planning.

Two-dimensional transthoracic echocardiography (2DE) is the mainstay of non-invasive anatomic and hemodynamic evaluation of PDAs. Three-dimensional echocardiography (3DE) allows for multiplanar interrogation of cardiac structures which can enhance visualization of complex anatomy [6–12]. Guidelines and a growing body of research exist for the utility of and optimal imaging by 3DE for multiple congenital cardiac anomalies, leading 3DE to become an increasingly important imaging modality for pre-interventional planning and intra-procedural guidance for several procedures in older children and adult patients [6, 10, 12–15]. These imaging guidelines and described use of 3DE in neonatal and pediatric procedural planning to date have not included the PDA.

Despite 3DE’s successes in aiding cardiac and vascular interventions in adults, there are very limited data on the precision and incremental benefit of transthoracic 3DE on assessing small pediatric vascular structures such as the PDA. The data that do exist, while encouraging, predominately involve older children greater than 1 year of age [16, 17], while many children who require a PDA intervention will need it at a younger age and smaller size.

Given the neonatal population is at particular risk of several complications of having a PDA and are frequently intervened on at a young age, 3D imaging of the patent ductus arteriosus may have strong clinical value in planning PDA procedures for review of multiplanar structural analysis prior to or during intervention. A foundational understanding of 3DE’s ability to precisely measure basic characteristics of the PDA is therefore needed; however, this is currently lacking in the current literature. In order to investigate this, we aimed to assess the association of 3DE measurements of the PDA as compared to clinical standard 2DE.

## Methods

### Study Design and Population

Subjects were enrolled prospectively between November 2020 and March 2022 at the University of California, San Francisco Benioff Children’s Hospital Mission Bay campus. The study consent and protocol were approved for use by the UCSF Institutional Review Board and informed consent for study image acquisition was obtained prior to imaging from a parent or guardian for subjects enrolled. Neonates and infants less than 1 year of age, greater than 32 weeks corrected gestational age, and greater than one kilogram with a known PDA as part of a structurally normal heart or with congenital heart disease were considered eligible for the study. Infants on oscillator ventilation, more than one vasoactive agent, and those deemed clinically unstable by the primary treatment team were excluded from participation. Collected demographic information included gestational and corrected gestational age, birth weight, current weight and length at time of study, mode of ventilation, and description of cardiac anatomic diagnosis. All infants imaged were in the neonatal intensive care unit or the pediatric cardiac intensive care unit at the time of enrollment and imaging.

### Image Acquisition

Study images were collected on the General Electric (GE) Vivid E95 ultrasound system (GE HealthCare, Chicago, IL) utilizing the GE 6S ultrasound transducer for 2D image acquisition and the GE 4 Vc-D matrix array 4D transducer for 3D volume acquisition. Three-beat EKG-gated images were collected with and without color Doppler from the parasternal short axis (PSAX) and suprasternal notch (SSN) positions using both the 2D and 3D probes. Narrowed sector width and zoom functions were used to take highest resolution images possible targeting the PDA. 3D acquisition was captured at a minimum of 20 frames-per-second for all studies and in the “FlexiView” and “HDLive” settings.

### Measurements

**Fig. 1:**
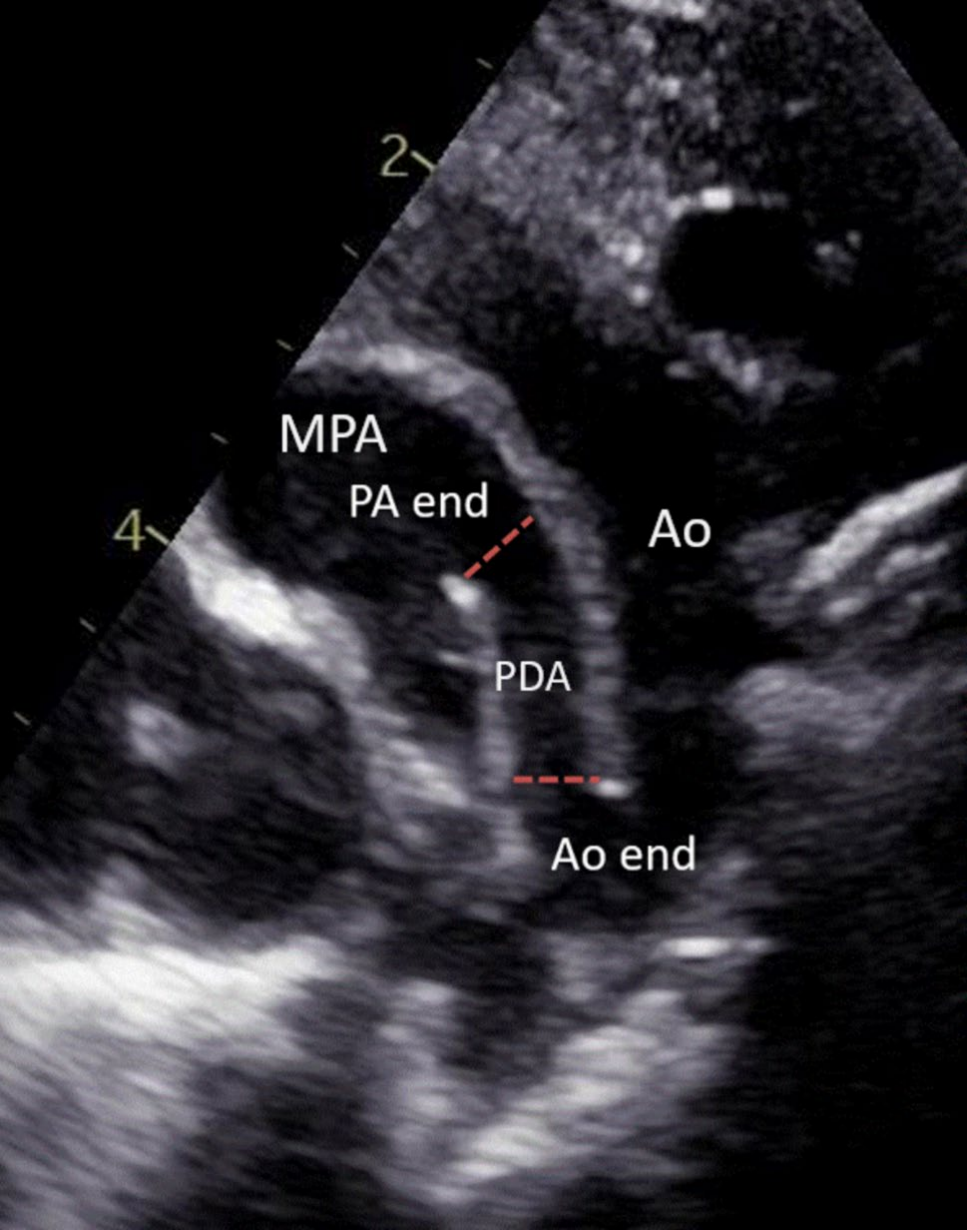
Example of measurement positions as described in Methods for a selected PDA image. Image taken from the suprasternal notch position, with the ductus between the two red dotted lines. *MPA* main pulmonary artery, *PA* pulmonary artery, *Ao* aorta

Two trained investigators (SJ and CS) conducted image acquisition and performed measurements, including multiplanar adjustment for 3D imaging. Measurements for selected images and post-processing were performed on the EchoPAC software system (GE HealthCare, Chicago, IL). Both readers first performed an unblinded calibration of four images at each view, at the aortic and pulmonic end, by 2DE and 3DE to ensure consistent measurement practices. After which, a previously selected subset of paired 2DE and 3DE images for each subject were reviewed and measured by each reader (SJ and CS) in a blinded fashion. All selected images were anonymized at time of image acquisition. For each image, the linear measurement of pulmonic and aortic end diameter of the PDA were recorded from the inner surfaces of the vessel lumen at 90 degrees to the vessel wall for consistency of measurement (Fig. 1). 3D volumes underwent multiplanar adjustment and signal-adjustment for image optimization prior to measurement.

### Statistical Analysis

Statistics were performed with STATA (version 17.0). Interobserver variability in 2DE and 3DE measurements for two readers was assessed by Bland–Altman assessment, as well as by interclass correlation (two-way, random effects) with 95% confidence interval for consistency of measurement, and coefficient of variability. Association of 2DE with 3DE measurements was assessed by Bland–Altman assessment, with differences in measure assessed by *t-*test.

## Results

Thirty subjects were enrolled for study participation, 29 of which had adequate image quality by 2DE and 3DE for measurement, one image set was corrupted and could not be interpreted. Subject characteristics are reported in Table 1.

**Table 1:** Patient demographics

% female	38%
Median gestational age, weeks (IQR)	37.6 (31.7–39.0)
Median corrected gestational age at date of study, weeks (IQR)	39.4 (38.3–41.7)
Median birthweight, kg (IQR)	2.960 (1.705–3.200)
Median weight at date of study, kg (IQR)	3.250 (2.870–4.295)
Median length, cm (IQR)	49.0 (48.0–52.2)
Mode of ventilation	
Room air	15 (52%)
Nasal cannula/high-flow nasal cannula	3 (10%)
CPAP/BiPAP	6 (21%)
Intubated	5 (17%)
Cardiac anatomy	
PDA, otherwise structurally normal, ± ASD/VSD	13
Left heart obstructive lesions	8
Right heart obstructive lesions	4
Transposition (dTGA ± VSD, Taussig-Bing)	4

*ASD* atrial septal defect, *VSD* ventricular septal defect

Median gestational age was 37.6 weeks (IQR 31.7–39.0), median corrected gestational age at the time of imaging was 39.4 weeks (IQR 38.3–41.7). Thirteen subjects had a PDA with normal segmental cardiac anatomy with or without atrial or ventricular septal defects (ASD/VSD), 8 had left heart obstructive lesions (aortic valve stenosis, HLHS, or aortic arch obstruction), and 4 had right heart obstructive lesions (pulmonary atresia with intact ventricular septum, Tetralogy of Fallot with pulmonary atresia, and severe Ebstein anomaly with pulmonary atresia). Four had transposition lesions (d-transposition of the great arteries (dTGA) ± VSD or Taussig-Bing anomaly). Median PDA sizes were variable based on position at PA or AO end and are summarized in Table 2.

**Table 2:** Summary of PDA sizes measured by 2DE and 3DE

		Median PDA size (mm) (IQR)
2D	SAX PA	4.42 (3.75–5.14)
	SAX Ao	4.72 (4.11–5.77)
	SSN PA	4.19 (3.53–4.91)
	SSN Ao	4.52 (3.92–5.76)
3D	SAX PA	4.31 (3.78–5.08)
	SAX Ao	4.79 (4.12–5.83)
	SSN PA	4.14 (3.70–5.10)
	SSN Ao	4.67 (3.99–5.78)

Interobserver variability assessments are summarized in Table 3. Interclass correlation for all 2DE and 3DE views was high with similarly strong limits of agreement, with all interclass correlation above 0.93, and coefficient of variation less than 10%. Bland–Altman assessment for all views showed low bias and generally narrow limits of agreement.

**Table 3:** Interobserver agreement metrics for measurements at each position by 2DE and 3DE

		ICC	Bland–Altman		Coefficient of
		Coefficient (95% LOA)	Bias (mm)	LOA	Variation (%)
2D	PSAX PA	0.962 (0.920–0.982)	− 0.103	(− 0.778–0.573)	7.52
	PSAX Ao	0.932 (0.836–0.970)	− 0.222	(− 1.098–0.653)	9.23
	SSN PA	0.977 (0.952–0.989)	− 0.070	(− 0.578–0.438)	5.88
	SSN Ao	0.963 (0.860–0.986)	− 0.200	(− 0.749–0.349)	5.89
3D	PSAX PA	0.966 (0.928–0.984)	− 0.003	(− 0.644–0.637)	7.12
	PSAX Ao	0.951 (0.845–0.981)	− 0.219	(− 0.887–0.450)	6.96
	SSN PA	0.953 (0.892–0.979)	− 0.157	(− 0.850–0.537)	7.85
	SSN Ao	0.944 (0.817–0.978)	− 0.244	(− 0.963–0.475)	7.52

ICC was high, with limited bias, and generally low variability in measurements

**Fig. 2:**
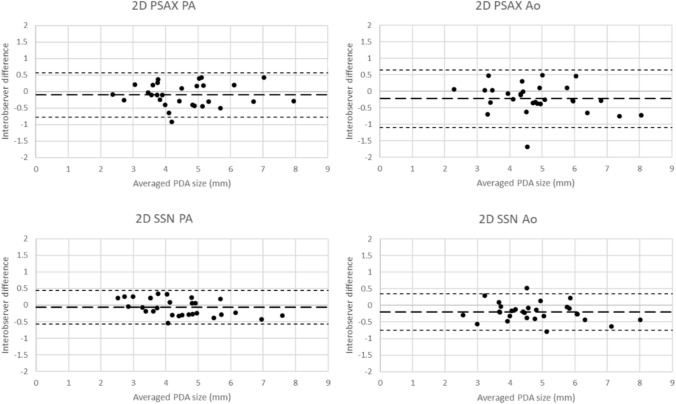
Interobserver Bland–Altman plots for 2D imaging for two observers in the views and positions utilized for PDA visualization. Data suggest good agreement between observers with generally narrow interobserver differences. Broad line indicating bias, fine dotted line indicating limit of agreement (LOA)

**Fig. 3:**
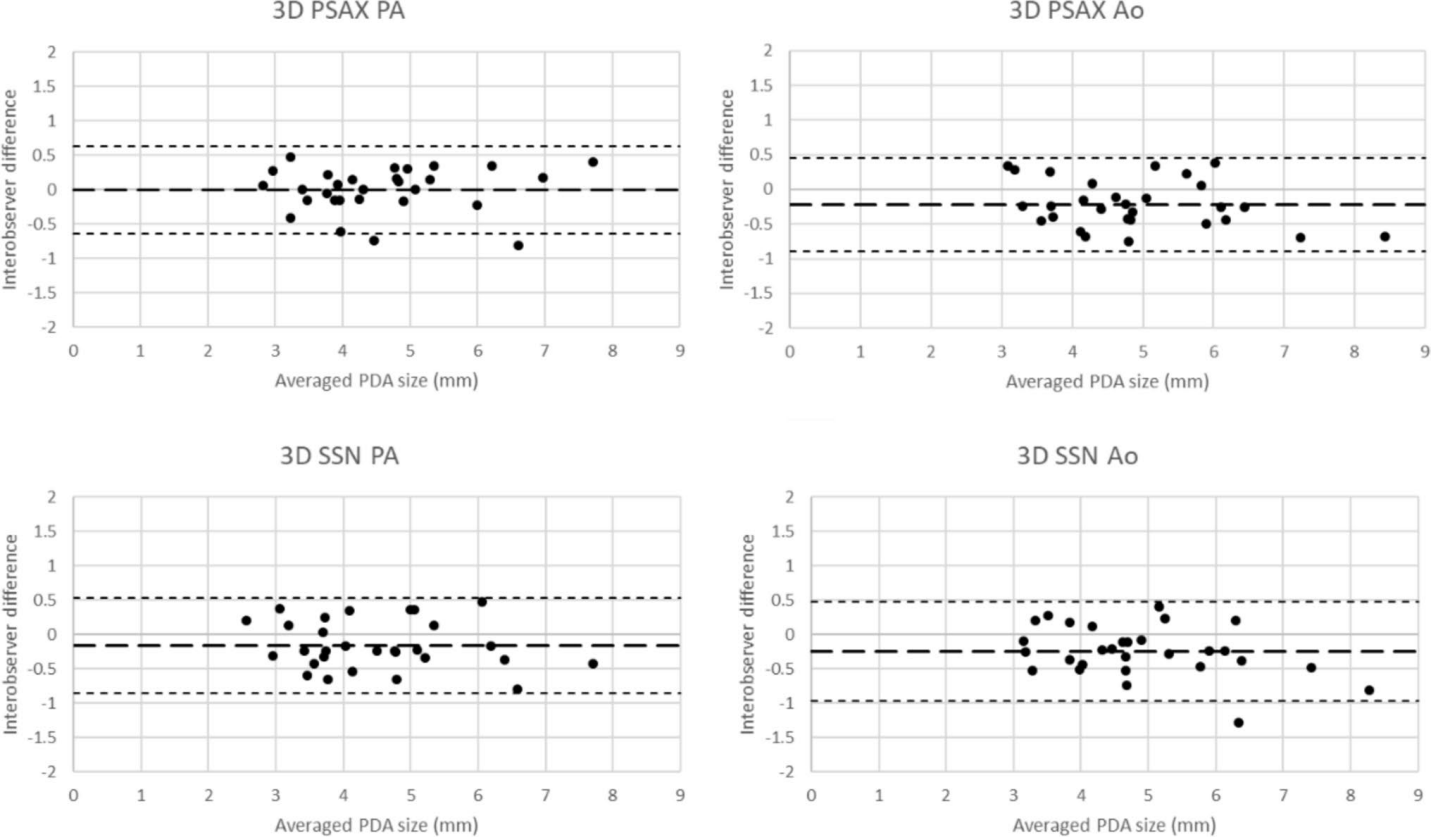
Interobserver Bland–Altman plots for 3D imaging for two observers in the views and positions utilized for PDA visualization. Data suggest good agreement between observers with generally small interobserver differences. Broad line indicating bias, fine dotted line indicating limit of agreement (LOA)

Bland–Altman for 2DE interobserver variability is demonstrated in Fig. 2, graphically demonstrating low bias and narrow limits of agreement (LOA) with similar findings for 3DE (Fig. 3) across the range of PDA sizes measured, with infrequent outliers. For each observer, there was a very slight bias generally toward smaller 2D measurement than 3D; however, this was only inconsistently statistically significant dependent on observer (Table 4).

**Table 4:** Bland–Altman assessment bias and limits of agreement, as well as *p*-value differences for 2DE compared to 3DE for both observers in respective views, demonstrating good agreement in measurement of 2DE compared with 3DE from the same view, with a very slight bias toward smaller 2D measurement which was inconsistently statistically significant dependent on the observer and plane

Observer 1:			
	Bland–Altman		
	Bias (mm)	LOA	*p*-value
PSAX PA	0.043	(− 0.554–0.641)	0.449
PSAX Ao	− 0.056	(− 0.832–0.719)	0.451
SSN PA	− 0.142	(− 0.763–0.478)	0.022
SSN Ao	− 0.149	(− 0.828–0.531)	0.029

Observer 2:			
	Bland–Altman		
	Bias (mm)	LOA	*p*-value
PSAX PA	− 0.056	(− 0.620–0.508)	0.317
PSAX Ao	− 0.060	(− 0.638–0.519)	0.277
SSN PA	− 0.056	(− 0.663–0.551)	0.329
SSN Ao	− 0.105	(− 0.710–0.500)	0.094

When comparing each observer’s 2DE measured values to 3DE, Bland–Altman analysis showed similarly strong agreement, limited bias, and generally low variability in measurement (Table 4).

## Discussion

Our findings indicate that 2DE and 3DE have strong and consistent agreement and precision in common measures of the aortic and pulmonary end of the PDA in neonates. Interobserver variability between two observers trained in use of and interpretation of 3DE for PDA assessment show good agreement in the views assessed, by both 2DE and 3DE. There was low bias and limited variability in measurements from 3DE views, and similarly strong agreement between paired 2DE and 3DE images. Importantly, the precision of measures was also generally narrow, with limits of agreement generally less than 1.0 mm in all views obtained, and when comparing 2DE to 3DE for comparable images.

From a technical standpoint, the data suggest that 3DE for assessment of small vascular structures is technically feasible and does not lead to loss of precision in neonatal subjects. We have demonstrated that through experience with acquisition, consistent application, and the utilization of simple image adjustments, it is feasible to attain high-resolution and precise measurements that are comparable to conventional imaging techniques.

**Fig. 4:**
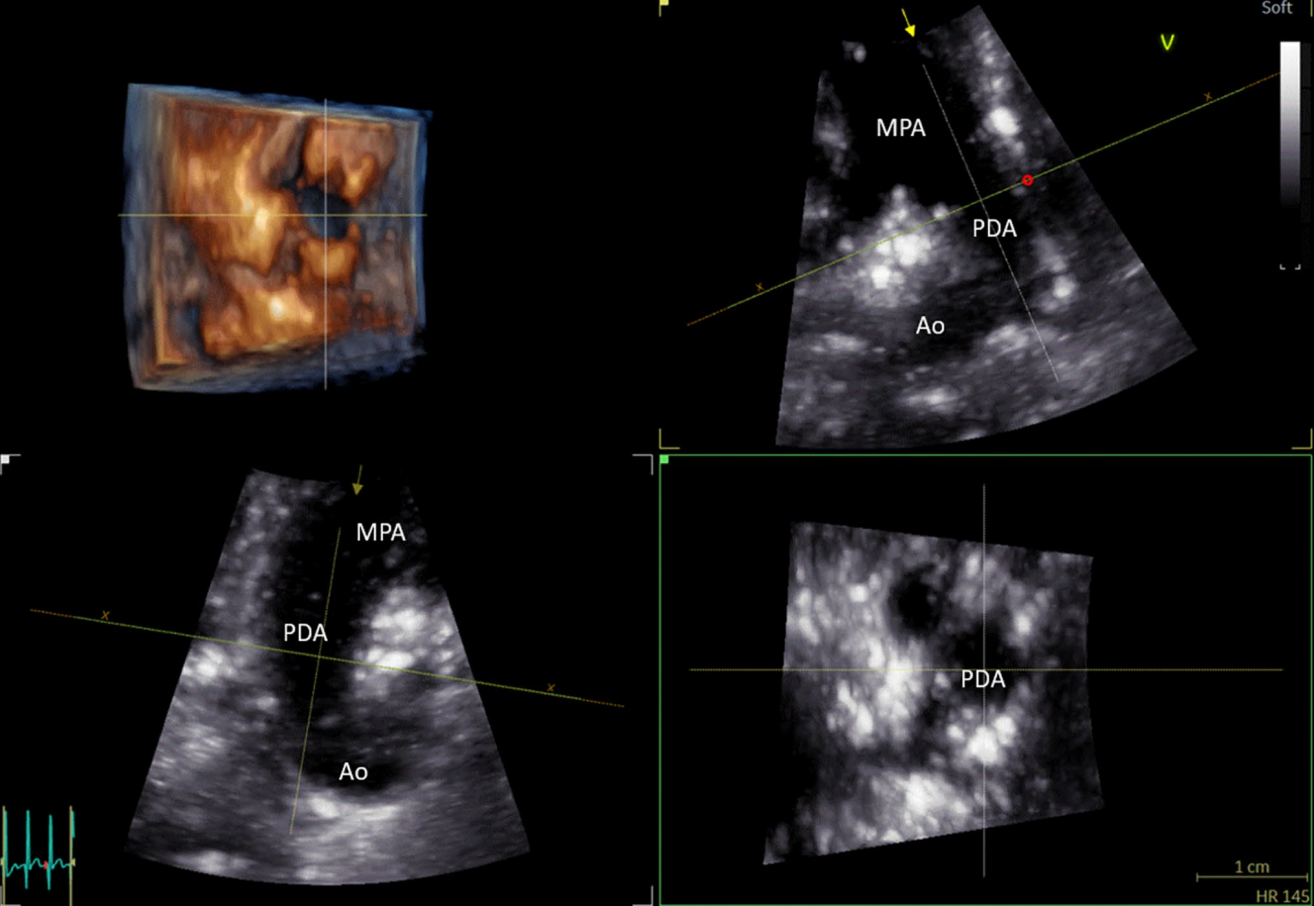
3D image of a large PDA with multiplanar and 3D representation. The 3D rendering of the PDA is seen in the top left corner, demonstrating a “down the tunnel” view of the PDA from the main pulmonary artery to the aorta. The top-right image represents the reference plane of the PDA from a parasternal short axis view. Individual axes have been adjusted to represent the full diameter of the PDA in each view

An added benefit of 3DE is its ability to utilize multiplanar viewing to increase precision and better understand 3D anatomy. For example, Fig. 4 demonstrates the 3DE imaging of a large PDA with a parasternal short axis view demonstrated in the upper right corner, with an axis line projecting down the PDA lumen. In the bottom right and bottom left corners are the axial and sagittal cross-section of the vessel lumen, respectively.

One advantage of utilizing a 3D volume is the position of the cross-section of the vessel lumen can be adjusted to avoid foreshortening the widest vessel diameter. While inconsistently statistically significant, the very slight bias toward larger PDA size with 3D imaging may be reflective of this, or that Observer 1’s imaging in particular in some planes may have benefitted from further multiplanar manipulation to fully “open” the vessel.

The clinical applications for 3DE in procedural planning have been demonstrated for several percutaneous interventions in adults, a notable example being the work by Fan et al. [14]. Here, the authors demonstrated that 3DE-derived datasets used for device sizing prior to left atrial appendage occlusion procedures led to a higher implantation success rate, shorter procedural time, and fewer devices per procedure than traditional intraoperative transesophageal echocardiography use. While the patient population and structure of interest in our study are inherently smaller in size, the clinical potential for 3DE in procedural planning for PDA interventions is analogous. A ductal closure device sized too large risks obstruction of the pulmonary artery at one end of the PDA, or of the aorta on the other. Conversely, an inappropriately small device increases risk of embolization [18–23].

While most cases of inappropriate sizing do not require surgical intervention, repeated placement and retrieval increases procedure time, radiation exposure, and nephrotoxic contrast exposure—risks which are more common and magnified in the premature and infant populations [18, 20, 23]. For these reasons, appropriate imaging and sizing of the ductus is of significant importance [19]. Beyond procedural risk, transportation of high-risk neonates between locations (i.e. to the cardiac cath lab) alone carries morbidity risk [24, 25]. In order to reduce exposure to radiation, contrast, and limit transport of such small infants, recent works have already investigated real-time, bedside, 2D echocardiogram-guided neonatal PDA procedures with limited to no contrast or fluoroscopy, demonstrating good procedural success in trained centers [23, 26, 27]. Recent work has also demonstrated that 2DE performed by skilled technicians demonstrates comparable measurement to angiography for infants and neonates undergoing PDA closure [28]. By extension, utilization of 3DE for procedural planning, and potentially intra-procedural guidance, may offer opportunities to improve success rates with PDA catheterization procedures, reduce procedural contrast and radiation exposure, limit risks of device embolization, and limit need for transport in this often fragile patient population.

While our study is aided by its prospective design, focus on neonates as the population of interest, and heterogeneous subject anatomy, an obvious limitation is a relatively small sample size. Not all patients in the study period with a PDA diagnosed by echocardiography at our center could be approached to participate in the study; rather, focus was made on those with known related heart disease and/or those more likely to undergo transcatheter PDA interventions in order to capture the population of greatest interest. As such, many of the PDA sizes imaged were larger and interobserver accuracy may not be as strong for tiny or small PDAs. Some, but not all, of the patients with isolated PDAs underwent transcatheter intervention, which limits our ability to compare to angiographic measurements in a meaningful way given the small sub-group size. Similarly, it is worth noting that imaging alone does not define a PDA as “hemodynamically significant,” which is beyond the scope of this paper.

While analogous clinical applications exist for 3DE, our data on their own do not prove clinical utility for neonatal PDA procedures. Once measurement agreement to current imaging standards—such as 2DE, computed tomography, and angiography—is proven, clinical utility of 3DE may then be assessed by post-hoc comparison of 3D-dataset-driven device placement to those performed in the catheterization lab. Comparison to multimodal imaging would also benefit from assessment of other PDA features, including ductal length, tortuosity, and ductal type. Finally, using 3D datasets for pre-procedural prediction of simulated placement of appropriate devices will determine clinical application.

## Conclusions

Our data suggest 2DE and 3DE have strong interobserver agreement and paired 2DE- 3DE images show low bias and limited variability when comparing common PDA measures. Further study should aim to compare 3DE agreement with other PDA imaging modalities, as well as expanding broader subject size, ductal size, and ductal type to help determine future potential utility of 3DE in PDA procedural planning.

## Data Availability

No datasets were generated or analysed during the current study.
